# Means of Recognising the Presence of Morphia in Cases of Poisoning by That Substance

**Published:** 1847-09

**Authors:** 


*Means of Recognising the Presence of Morphia in Cases of Poisoning by that Substance.*—This method is detailed in the Gazette Médicale, as proposed by M. Mermu. The solid matters ejected from the stomach are first to be washed with water, slightly acidulated with acetic acid. The product of the several washings is to be mixed with any liquids which can be collected. If one has liquids only at his disposal, those are to be mixed with the water, acidulated as above. In either case, the resulting mixture is then to be evaporated to dryness, and treated with boiling alcohol, to separate the animal matters. To the alcoholic liquid, previously filtered, some tincture of gall-nuts is to be added—the tincture being made of 125 parts of alcohol with 250 parts of coarsely powdered gall-nuts; and the whole to be let digest fifteen days, which will precipitate the little animal matter dissolved by the alcohol, and the combination of tannin and morphia thence resulting will remain in solution.

The liquid is next to be filtered, mixed with a small quantity of distilled water, and then a solution of gelatine to be added in excess, in order to decompose the tannate of morphia. The morphia having gone over to the gelatine, the tannin with which it was previously in combination becomes dissolved by the spirit. To separate the precipitate of tannin and of gelatine, it is necessary again to filter; and when the alcohol is evaporated, the morphia will be left, which may be recognized by its particular tests.—*London Lancet.*

